# Epidural analgesia reduces perioperative myocardial infarction and all-cause mortality after cardiac surgery: but at least 25 epidural hematomas have already happened

**DOI:** 10.1186/cc13612

**Published:** 2014-03-17

**Authors:** G Landoni, M Greco, I Francesca, D Taddeo, O Saleh, A Belletti, A Putzu, R Lembo, A Zangrillo

**Affiliations:** 1 Vita-Salute San Raffaele University, Milan, Italy

## Introduction

Epidural analgesia on top of general anesthesia in cardiac surgery might improve relevant clinical outcomes but the incidence of epidural hematoma is under-reported.

## Methods

An international web-based, online, viral, anonymous survey, a systematic review of the literature, and a meta-analysis of randomized and matched studies were performed.

## Results

Nine epidural hematomas were identified in 198 published manuscripts. The risk of epidural hematoma (9:13,429) calculated on all published evidence might be therefore estimated to be 1:1,492 (95% confidence interval (CI) = 1:2,857 to 1:833). Through an anonymous, web-based, viral, international survey, we identified at least 16 further nonpublished epidural hematomas together with at least 72,400 epidural analgesia catheters positioned in cardiac surgery in the last 20 years. The risk of epidural hematoma (25:85,829) is therefore 1:3,436 (95% CI = 1:2,325 to 1:5,076) including both published and unpublished data. Out of the 66 randomized and case-matched studies, 57 trials reported the incidence of all-cause mortality at the longest available follow-up with a significant reduction in the epidural group (59/3,137 (1.9%) in the epidural group vs. 108/3,246 (3.3%) in the control arm, RR 0.64 (95% CI 0.48 to 0.85), *P *= 0.002, NNT = 69).

## Conclusion

Epidural analgesia on top of general anesthesia in cardiac surgery might reduce the incidence of all-cause mortality (NNT 69). The incidence of epidural hematoma in this setting is 1:3,436 (95% CI = 1:2,325 to 1:5,076) including both published and unpublished data. In fact, we identified at least 25 epidural hematomas that occurred so far from the following countries: Belgium (*n *= 1), Brazil (*n *= 1), France (*n *= 1), Germany (*n *= 2), India (*n *= 2), Italy (*n *= 1), Japan (*n *= 2), Korea (*n *= 1), Malaysia (*n *= 1), Norway (*n *= 2), Russia (*n *= 3), Sweden (*n *= 1), the UK (*n *= 3), and the USA (*n *= 4). Even if from the public health point of view the benefits seem to encourage the use of epidural analgesia in cardiac surgery with a possible reduction in perioperative mortality, this topic merits further investigation and the decision to insert the epidural catheter should be discussed with the patient considering both local experience and legal dispute in case of medical complications.

